# O056: The introduction of a surgical site infection prevention bundle on a nationwide scale

**DOI:** 10.1186/2047-2994-2-S1-O56

**Published:** 2013-06-20

**Authors:** T Hopmans, L Soetens, J Wille, P van den Broek, M Koek, B van Benthem

**Affiliations:** 1 Epidemiology and Surveillance, National Institute of Public Health and the Environment, Bilthoven, The Netherlands; 2 Department of Infectious Diseases, Leiden University Medical Centre, Leiden, The Netherlands

## Introduction

In 2009 a patient safety program, consisting of a 4-item surgical site infection (SSI)-prevention-bundle, was introduced in the Netherlands. The infection prevention bundle consist of: timely antibiotic prophylaxis, no preoperative surgical site hair removal, perioperative normothermia and hygiene discipline on the OR.

## Objectives

The aim of the SSI-bundle introduced by the safety program is to reduce the SSI-rate through creating more awareness about the importance of patient safety among hospital employees. The objective is a 90% compliance with the complete SSI-prevention bundle.

## Methods

Data collection (2009-2012) was incorporated in the PREZIES surveillance network for healthcare associated infections. Compliance with the four interventions was registered separately and combined in patients, who underwent a surgical procedure present on the list of indicator procedures ( a selection of 13 procedures of 6 different specialties). Log binomial regression analysis was used to calculate relative risks (chance) on compliance, stratified by medical specialty, calendar time and participation period.

## Results

Registration of the complete bundle was around 20% by the end of 2011 (varying between 28% and 66% for the individual bundle items). Compliance with the individual bundle items increased over time: by the end of 2011, three out of four items reached a compliance greater than 75%. However, compliance with the complete bundle reached 27%.

## Conclusion

This is the first patient safety program implementing a SSI-bundle on a nationwide scale. The objective of 90% compliance with the complete bundle was not met; although compliance increased over time, it remained low. Likewise, registration did not exceed 20%. We recommend to prolong this program, however the implementation process must be strengthened. A qualitative study is suggested to gain insight in barriers of this process.

## Disclosure of interest

None declared.

